# Induction of Brain Microvascular Endothelial Cell Urokinase Expression by *Cryptococcus neoformans* Facilitates Blood-Brain Barrier Invasion

**DOI:** 10.1371/journal.pone.0049402

**Published:** 2012-11-08

**Authors:** Jamal Stie, Deborah Fox

**Affiliations:** 1 Research Institute for Children, Louisiana State University Health Sciences Center, New Orleans, Louisiana, United States of America; 2 Department of Pediatrics, Louisiana State University Health Sciences Center, New Orleans, Louisiana, United States of America; Leibniz Institute for Natural Products Research and Infection Biology- Hans Knoell Institute, Germany

## Abstract

The invasive ability of the blood-borne fungal pathogen *Cryptococcus neoformans* can be enhanced through interactions with host plasma components, such as plasminogen. Previously we showed by *in vitro* studies that plasminogen coats the surface of *C. neoformans* and is converted to the active serine protease, plasmin, by host plasminogen activators. Viable, but not formaldehyde- or sodium azide-killed, cryptococcal strains undergo brain microvascular endothelial cell-dependent plasminogen-to-plasmin activation, which results in enhanced, plasmin-dependent cryptococcal invasion of primary bovine brain microvascular endothelial cells and fungal ability to degrade plasmin substrates. In the present work, brain microvascular endothelial cells cultured with viable, but not killed, cryptococcal strains led to significant increases in both urokinase mRNA transcription and cell-associated urokinase protein expression. Soluble urokinase was also detected in conditioned medium from brain microvascular endothelial cells cultured with viable, but not killed, *C. neoformans*. Exposure of plasminogen pre-coated viable *C. neoformans* to conditioned medium from strain-matched brain microvascular endothelial cell-fungal co-cultures resulted in plasminogen-to-plasmin activation and plasmin-dependent cryptococcal invasion. siRNA-mediated silencing of urokinase gene expression or the use of specific inhibitors of urokinase activity abrogated both plasminogen-to-plasmin activation on *C. neoformans* and cryptococcal-brain microvascular endothelial cell invasion. Our results suggest that pathogen exploitation of the host urokinase-plasmin(ogen) system may contribute to *C. neoformans* virulence during invasive cryptococcosis.

## Introduction

*Cryptococcus neoformans* is an encapsulated, facultative intracellular pathogen that is globally distributed and recognized as a leading cause of fungal meningitis in immunocompromised people [1], [2]. Approximately one million new cases of cryptococcosis are reported annually and result in an estimated 600,000 deaths [3]. The primary means of cryptococcal-host entry is through the inhalation of fungal conidia and/or desiccated yeast forms, which can lodge and germinate within alveolar tissue [4]. Cryptococcosis is effectively countered by a T cell-dependent proinflammatory immune response and is regarded as an AIDS-defining illness in patients with severe CD4+ T-cell lymphocytopenia [≤100 cells/cm^3^; [3], [5], [6]]. In the absence of protective T cell immunity, *C. neoformans* can readily disseminate from the lungs into the CNS by a hematogenous route, where, if left untreated, leads to patient death.

*C. neoformans* can parasitize macrophages that traffic between the blood and tissues and eventually escape from the phagocytes into tissues or organs, such as the CNS, by a phagosomal extrusion process without affecting host cell viability [7], [8]. Alternatively, blood-borne *C. neoformans* can directly invade the blood-brain barrier (BBB) via paracellular passage between brain microvascular endothelial cells (BMEC), or by a transcellular mechanism that is dependent on fungal cell internalization. Specific interactions between *C. neoformans* and BMEC result in apical-to-basal transcytosis of the fungal pathogen [9]–[14]. Fungal-BMEC transcytosis is mediated by the hyaluronic acid (HA) component of the *C. neoformans* capsule structure [15], an essential virulence factor of *C. neoformans* [16]. Cryptococcal-BBB invasion by a paracellular route occurs in connection with either damage to the brain microvasculature [17]–[21] or focused proteolytic degradation of inter-endothelial junctions [21].

Proteolytic degradation of intercellular junctions in tissues is a routine physiological process mediated by functionally specialized host proteases of the plasminogen-fibrinolytic system, which consists of multiple protease families [22]. Proteolytic activation of the central zymogen, plasminogen, to the serine protease, plasmin, initiates a complex cascade of interactions that mediate fibrinolysis required for vascular homeostasis, or tissue degradation and remodeling required for immune function as well as tissue growth, development and repair processes [23]. Urokinase is an inducible, host-derived plasminogen(-to-plasmin) activator (PA) that can facilitate plasmin's function in immunological processes, such as immune cell recruitment [24], and also contributes to plasmin-dependent microbial invasion of host tissues [25], including the CNS [26]. Like plasmin, urokinase is expressed in zymogen form, where activation entails proteolytic conversion of a single-chain proenzyme to a disulphide-linked, two-chained serine protease [27], [28]. This activation process is amplified by a feedback activation loop between plasmin and urokinase on cell surfaces that concentrates proteolytic activity at intercellular or cell-matrix interfaces [29].

Whereas urokinase is a plasminogen-specific serine protease, plasmin is a broad-spectrum protease that degrades fibrin-enriched extracellular matrices and basement membranes, in addition to activating other zymogen protease systems [22]. The central downstream effectors of plasmin include matrix metalloproteinases (MMPs) and procollagenases that enhance vascular permeability by the focalized degradation of inter-endothelial junctions and cell-associated protein networks, respectively [22]. Plasmin and urokinase therefore function as regulators of a rapidly accessible and potent reservoir of extracellular proteases that can potentially serve protective versus pathologic roles during the innate immune response by alternatively facilitating the containment and/or dissemination of infectious pathogens, respectively.

Plasminogen attachment to cells or fibrin matrices is essential for plasminogen-to-plasmin activation [30]. Plasminogen primarily binds C-terminal lysine residues, a common constituent of surface-localized proteins on host tissues, and many eukaryotic and prokaryotic pathogens [30]. Several fungal pathogens, including *C. neoformans*, *Paracoccidioides brasiliensis* and *Candida albicans*, exhibit plasminogen binding activity *in vitro* [31]–[34]. Certain plasminogen binding bacterial pathogens, such as *Yersina pestis* and group A streptococci, additionally express endogenous PA activities that mediate their dissemination in animals [35]–[37]. Pathogens can alternatively use host PA for plasminogen-to-plasmin activation on their cell surface. For example, *Helicobacter pylori*-induced urokinase expression in gastric mucosa correlates with invasive pathogenesis [25], [38]. Similarly, induction of host PA expression by *Borrelia burgdorferi* enhances plasmin-dependent BMEC invasion, *in vitro* [26]. In accord with these findings, we have shown that fungal-BMEC interactions *in vitro* potentiate the plasmin-dependent invasive ability of *C. neoformans*, thus suggesting a role for BMEC-PA expression during cryptococcosis [21].

The importance of urokinase expression during pulmonary cryptococcosis is well established. Urokinase knockout animals fail to mount an effective pulmonary immune response to *C. neoformans*, leading to uncontrolled infection [39]. The absence of urokinase results in impaired leukocyte recruitment and macrophage antimicrobial activity during pulmonary cryptococcosis, and the loss of Th1 cytokine dominance within regional lymph nodes relative to wild-type animals infected with *C. neoformans* [39], [40]. The defective immune response of urokinase knockout animals is further evidenced by impairments in cellular proliferation and proinflammatory cytokine secretion by T-lymphocytes after *C. neoformans* re-challenge, *in vitro*. Urokinase-null animals also exhibit greater infiltration of *C. neoformans* into major organs, such as the spleen and brain, and have markedly reduced survival times [39]. These results show that urokinase is essential for the protective pulmonary immune response against *C. neoformans* and subsequent containment of infection to the lungs. However, a direct, facilitating role for urokinase in cryptococcal-host invasion during disseminated cryptococcosis has not been addressed.

In addition to its role in host immune function, urokinase can directly promote pathogen dissemination by catalyzing plasminogen-to-plasmin activation on microbial surfaces in association with plasmin-dependent pathogen-host invasion. As *C. neoformans* binds plasminogen, *in vitro*, we examined whether this pathogen could additionally subvert the function of urokinase to facilitate plasmin-dependent fungal invasion of host tissues. In this report, we use a well-established BBB model system to show that *C. neoformans* induces urokinase expression in BMEC and that cryptococcal-induced BMEC urokinase expression is essential for fungal invasion of a BBB model. We suggest that *C. neoformans*-BMEC interactions induce plasmin- and urokinase-dependent changes in BMEC phenotype that cultivate a pro-invasive microenvironment during the blood-borne-dissemination phase of cryptococcosis.

## Materials and Methods

### Strains

The fungal strains used in this study include *C. neoformans* serotype D strains JEC21 (*MAT*α) and B3501A (*MAT*α) and the serotype A strain C23 [41]. *Saccharomyces cerevisiae* strain YPH499 was used as a non-invasive control. Strain FCH78 (*cap59::nat*) was generated by insertional mutagenesis of strain JEC21 as previously described [32].

### Fungal cell culture, preparation and counts

Strains were cultured to log phase in yeast extract-peptone-dextrose (YPD) medium at 25°C. Log phase *C. neoformans* strains were identified as thinly encapsulated by the absence of visible capsule on cells stained with India ink. An acapsular mutant, FCH78, tended to self-aggregate during preparation, and this was effectively addressed by successive suspensions in either PBS with 2.5% BSA or conditioned medium from uninfected 24 h BMEC cultures. Prior to assays, strains were washed three times in PBS with 1.5% BSA (PBS-BSA) and counted by use of a hemocytometer. Chemically-killed strains were prepared with either 3.5% formaldehyde or 10 mM sodium azide, as described previously [21].

### Plasmin(ogen) pre-coating of fungi

Fungal cells (10^8^) were coated with 50 µg purified human plasminogen (Glu-plasminogen, Fitzgerald Industries) in 500 µl PBS with 1.5% BSA at 37°C for 1 hr, and washed twice in cold PBS-BSA. Plasmin pre-coated yeasts were generated by incubating plasminogen coated cells with 5 µg tissue-type plasminogen activator (Calbiochem) as above for 4 h. Plasmin activity was verified in colorimetric assays using the plasmin-specific substrate, Chromogenix (Fisher Scientific) as described below.

### Inhibitor treatments

UPA-STOP (American Diagnostica) and amiloride (Calbiochem) were added at final concentrations of 100 nM and 15 µM, respectively, to transwells 5 min before fungal cell addition and the start of invasion assays. Fungal cells cultured for 12 h in the presence of working concentrations of each inhibitor described above exhibited no discernible changes in viability, growth rate, or confluent growth by TEER analysis. The protease inhibitor, aprotinin (Sigma), was added to assay medium at a concentration of 100 µg/mL prior to invasion assays, where indicated. Alternatively, BMEC cultures were treated with 5 µg/mL actinomycin D for 6 h prior to culture with *C. neoformans*. BMEC cell viability was not affected by actinomycin D treatment as determined by trypan blue exclusion.

### Plasmin activity assays

Activity assays were performed with the plasmin-specific synthetic substrate, Chromogenix or fibrinogen (Sigma). Chromogenix (S-2251, D-valyl-leucyl-lysine-p-nitroanilide dihydrochloride) was used at a final concentration of 150 mg/well in reaction buffer (0.32 M Tris-HCl, 1.77 M NaCl, pH 7.5) and included in microtiter plate wells containing 10^5^ fungal cells. Absorbance was read at 405 nm after 1 h incubation at 37°C. The presence of cell surface plasmin on yeast forms is indicated by the color change of medium supernatants, and the quantification of this color change at wavelength 405 nm was used for statistical measurements. Alternatively, fungal cells were suspended in reaction buffer (20 mM Tris-HCl, pH 7.6, 150 mM NaCl) containing 500 µg/mL fibrinogen (Sigma). Reaction volumes of 100 µL in 1.5 mL tubes were incubated 6 h at 37°C. Fungal cells were removed from reactions by sedimentation and the assay supernatants examined by SDS-PAGE followed by Western analysis.

### Endothelial cell culture

Primary bovine brain microvascular endothelial cells (BMEC) were purchased (Cell Applications, Inc.) either at second passage in cryopreserved ampules or at third passage as confluent cells in FBS-supplemented bovine brain endothelial cell medium (Cell Applications, Inc.) containing the anti-fungal agent, amphotericin B. Cultures were grown at 37°C with 5% CO_2_ in filter-top flasks (Corning) pre-coated with an attachment factor solution (Cell Applications, Inc) and subcultured as recommended by the supplier. BMEC cultures were harvested at or before passage six and verified for normal cell morphology, growth kinetics, confluence, and viability by Trypan blue exclusion before use in assays (data not shown).

### Endothelial barrier resistance

BMEC monolayer integrity and confluent growth in transwell inserts was confirmed by trans-endothelial electrical resistance (TEER) analysis using the Millicell electrical resistance system (Millipore). Prior to measurement, the culture medium of both upper and lower chambers of the transwells was replaced with 700 µL and 500 µL Hanks' balanced saline solution supplemented with 1.6 mM calcium and 0.9 mM magnesium (HBSS+). Transwell cultures were allowed to equilibrate for 30 min prior to TEER measurements across monolayers, according to manufacturer guidelines. Maximum resistance was typically observed four days after the seeding of BMECs in transwell inserts and persisted for at least 7 additional days. The barrier function of BMEC cultivated in transwell inserts was confirmed by TEER for maximal resistance prior to each assay. Background TEER values consisted of resistance measurements across transwell inserts without BMEC and were subtracted from the values reported in this study.

### Paracellular permeability measurement

The permeability of BMEC to FITC-dextran of average molecular weight 40 kD (Sigma Aldrich) was examined to further verify the integrity of BMEC monolayer growth in transwells. BMEC were prepared for tracer analysis by replacing culture medium from the upper and lower wells of transwells with 150 µL and 700 µL HBSS, respectively, in order to minimize the influence of hydrostatic pressure on the otherwise free-diffusion of tracer molecules (Quan & Godfrey, 1998). Changes in BMEC monolayer permeability were examined based on a previously described method (Wong & Gumbiner, 1997) with several modifications. Transwell inserts received 50 µg/mL FITC-dextran and allowed to equilibrate at 25°C under gentle agitation for 30 min. After 30 min, medium from the lower transwell chambers was collected at 15 min intervals over the course of 1 h. Transient changes in hydrostatic pressure between transwell chambers were avoided by using separate transwells for each measurement. FITC-Dextran present in medium collected from lower wells was measured at excitation and emission wavelengths of 488 nm and 520 nm, respectively using a single-channel Bio-Tek Synergy HT fluorescence microtiter plate reader. Signal was quantified as moles FITC-dextran from a standard concentration curve of tracer and as a percentage of the total FITC-dextran added to upper wells.

### Transwell invasion assays

Transwell assays were performed in 24-well Matrigel™ Invasion Chambers, an *in vitro* system for the study of cell invasion across BD Falcon™ cell culture inserts, each containing an 8 µm pore-size PET membrane coated with a uniform layer of BD Matrigel™ Basement Membrane Matrix (BD Biosciences). Matrigel™ is derived from tumor cell exudates and enriched in latent growth factors, cytokines and other zymogens. Transwells were prepared for use in functional assays as recommended by the manufacturer. Transwell inserts containing Matrigel were further coated with an attachment factor solution (Cell Applications) for 30 min at 37°C with 5% CO_2_, and seeded with BMECs at 10^5^ cells per transwell in 500 µl culture medium, with 700 µl added to lower chambers. Confluent growth was then monitored by the methods described above.

BMEC monolayer development required 4–5 days of growth after seeding to reach maximum resistance by TEER, after which the BMEC transwells were used for invasion assays. The assay medium used for transwell invasion assays included culture medium without amphotericin B containing plasminogen-depleted FBS. Plasminogen present in FBS was removed by immunoprecipitation with plasminogen polyclonal antibody (Fitzgerald Industries) covalently linked to CNBr-activated agarose beads (GE Healthcare) and used as recommended by the manufacturer (data not shown). BMEC-transwells were prepared for analysis by rinsing twice with 800 µL filter-sterilized HBSS to remove residual serum factors from transwell inserts prior the addition of fungal cells. 10^5^ fungal cells were used for transwell invasion experiments and pre-coated with plasminogen before assays as indicated. For siRNA experiments, BMEC were used for invasion assays 72 h after siRNA transfection. Transmigrated fungal cells were quantified from the lower chamber of transwell plates after 12 h incubation at 37°C with 5% CO_2_ and colony counts were determined from growth on YPD agar. BMEC cultures were confirmed for confluent growth by TEER prior to invasion experiments.

### Conditioned medium (CM) preparation

For the production of conditioned medium (CM), BMEC monolayers were grown to confluence in 100-mm plastic dishes (Corning) in routine culture medium (see above) at 37°C with 5% CO_2_, with confluent growth confirmed by phase microscopy. Confluent cultures were rinsed three times with FBS- and amphotericin B-free culture medium to remove serum proteins and incubation was continued in FBS- and amphotericin B-free culture medium for 12 h with the indicated viable or killed fungal strains at a 3∶1 multiplicity of infection (MOI). After 12 h, the CM was isolated and cleared of cellular debris by centrifugation at 3000× g for 5 min. The cleared CM was used to suspend fresh yeast cultures of the same strain as used in BMEC co-culture to produce the CM. Strains were pre-coated with plasminogen prior to suspension in CM at a final concentration of 10^8^/mL. The CM-suspended strains were incubated 4 h in a 37°C water bath and either solubilized in SDS-PAGE for Western blot analysis or used for transwell invasion analysis. Alternatively, CM was used for immunoprecipitation and fibrin overlay zymography as described below.

### Flow cytometry

Confluent BMEC were cultured in the presence or absence of viable or killed *C. neoformans* serotype A strain, C23, for 12 h at 37°C with 5% CO_2_ in plasminogen-depleted assay medium. After incubation, BMEC were washed extensively in 6-changes of HBSS (5 min/wash) with gentle agitation to remove yeast forms. BMEC were harvested in 20 mM EDTA in PBS without trypsin using a cell-scraper, to avoid protein loss, and transferred to suspension buffer [PBS, 200 units/mL aprotinin (Sigma), 44 µg/mL PMSF (MP Biomedical)], and one tablet of Complete protease inhibitor mix (Roche Diagnostics) at 4°C. Cells were counted by hemocytometer, sedimented at 250× g for 5 min, and suspended in 100 µL of PBS at 10^5^ cells/mL with 2 µg rabbit anti-urokinase polyclonal antibody or secondary antibody alone (see below) for 30 min on ice with intermittent agitation of reactions during incubation. Reactions were terminated by the addition of 100-fold excess (v/v) ice-cold PBS without antibody, centrifuged as above and suspended in the same buffer with 8 µg/mL Alexa Fluor chicken anti-rabbit 594-IgG conjugate and incubated as above. Reactions with secondary antibody were terminated as above and 10,000 cells per sample were examined for cell-associated fluorescence in a FACScan flow cytometer (Becton Dickinson) using FlowJo software. BMEC populations were gated for uniform size and density prior to assessing urokinase expression. Eighty percent to 95% of the 10,000 total cells examined included in post-experimental analysis, with the remaining cells excluded due to anomalous forward and/or side scatter profiles possibly from host cell damage during assays or sample preparation.

### Indirect immunomicroscopy

Confluent BMEC monolayers were developed on No. 2, 24×30 mm glass coverslips (Fisher Scientific) pre-coated with adhesion factor solution (Cell Applications) and cultured with or without *C. neoformans* strain C23 at 37°C with 5% CO_2_ in plasminogen-depleted assay medium. After 12 h, BMEC were extensively washed with 6×5 min changes in HBSS followed by fixation with 2% paraformaldehyde in PBS for 1 h at 4°C. Cells were washed twice in PBS containing 0.1% bovine serum albumin and stained with 10 µg/mL of anti-urokinase rabbit polyclonal antibody for 1 h at 25°C in the same buffer under gentle agitation. Cells were similarly washed and stained with 4 µg/mL Alexa Fluor chicken anti-rabbit 594-IgG conjugate, washed, mounted with SlowFade Gold antifade reagent with DAPI (Invitrogen) and examined for fluorescence using Olympus Bx51 or Zeiss LSM 510 microscopes; images were captured by a CCD camera and analyzed with MagnaFire SP (Olympus) or AxioVision (Zeiss) software. For some experiments, urokinase was selectively stripped from the BMEC surface by treatment for 3 min with 50 mM glycine HCl buffer, pH 3.0, in 0.1 M NaCl at 25°C, as previously described [42]. The acid-treated cells were rapidly equilibrated in HBSS and stained for urokinase expression as described above. For studies examining the efficacy of siRNA transfection, 7 h post-transfected and mock-transfected BMEC were washed 3×5 min washes in HBSS, prepared and mounted as above, and directly examined for the presence of Alexa Fluor 488-conjugated siRNA. The percent efficiency of siRNA delivery was determined as the number of fluorescent cells per total cells per field ×100%, with the average percentage reported from a total of 200 fields from 4 experiments (50 fields per experiment).

### Immunoprecipitation

Confluent BMEC were cultured with yeast forms from the indicated fungal strains at a 3∶1 MOI for 12 h at 37°C with 5% CO_2_. Monocultured BMEC were alternatively stimulated for 12 h with 20 nM phorbol myristate acetate (PMA) to positively control for urokinase expression [43]. DMSO was used to solubilize PMA and present in the medium of positive control cultures at a concentration of 0.01%. The negative control cultures [PMA(−)] that are paired with PMA(+) cultures in Results included 0.01% DMSO to control for the effects of DMSO. CM was collected from 12 h fungal-BMEC co-cultures and monocultured BMEC controls and centrifuged at 3000× g to remove free yeast cells and cellular debris. The cell-free CM was concentrated to 30 kD MWCO with Amicon Ultra filtration units (Sigma), supplemented with 1 mL PBS containing Complete protease inhibitor mix (Roche Diagnostics), and dialyzed for 24 h against 2-changes of 2 L PBS, pH 7.4, at 4°C. The dialyzed CM was supplemented with 1% Triton X-100 and the protein concentration determined using the bicinchoninic acid protein assay (Fisher Scientific). 0.5 mg/ml of CM was pre-cleared by adding unconjugated CNBr-activated Sepharose 4B (GE Healthcare) at a bead∶CM ratio 1∶2 (v/v) and incubating end-over-end for 1 h at 25°C. Sepharose was removed by 10,000 g sedimentation for 1 min in an accuSpin Micro R (Fisher Scientific). The pre-cleared CM was incubated as above with anti-urokinase polyclonal antibody (Santa Cruz Biotechnology) directly conjugated to CNBr Sepharose 4B according to manufacturer's instructions. Immunoprecipitates were washed 3×5 min in the same buffer at 25°C, eluted in SDS-PAGE buffer and prepared for analysis as described below.

For cellular urokinase immunoprecipitation, after fungal co-culture, BMEC were washed in 6-changes of HBSS at 25°C to remove adherent fungal cells (5 min/wash) with gentle agitation. The washed BMEC monolayers were directly solubilized in lysis buffer (PBS, pH 7.2, 0.1% SDS, 0.5% deoxycholic acid, 1% Triton X-100, 1 mM phenylmethylsulfonyl fluoride, 1 mM EDTA, and one tablet of Complete protease inhibitor mix) and mixed by rotation (Roto Mixer, Barnstead Thermolyne) for 30 min on ice. Cellular debris was removed from culture dishes with a cell scraper, and lysates were passed 6 times through a 21 gauge syringe, transferred to conical tubes and incubated an additional 30 min on ice with vortexing (5–10 s) every 10 min. Post-nuclear supernatants were obtained by centrifuging lysates at 10,000 g for 10 min to remove insoluble material and subsequently diluted to 1.0 mg/ml total protein and pre-cleared with CNBr Sepharose 4B as described above. Immunoprecipitation with anti-urokinase-conjugated CNBr Sepharose 4B and washes were performed as described above, and SDS-PAGE and Western analysis were as described below.

### Fibrin overlay zymography

The CM was examined for plasminogen activator activity based on a previously described technique [44] with several modifications. CM samples were dissolved in SDS-PAGE buffer without heating and fractionated by SDS-PAGE under non-reducing conditions. Fibrin indicator agarose gels were prepared by the addition of 28 mg fibrinogen in 5 mL pre-warmed (37°C) PBS, gradually added over 2 h to prevent flocculation. The fibrinogen-PBS solution was mixed with 3 units of thrombin protease and immediately added to 1% (w/v) melted agarose (56°C) in PBS and poured onto Gelbond support film to prepare the fibrin indicator gels. SDS-PAGE gels were renatured by 1 h incubation in 2.5% Triton X-100, rinsed several times with sterile-filtered water, and overlaid onto the fibrin-indicator gel and incubated within a humidified chamber at 37°C for 20 h. After incubation, the SDS-PAGE gel was discarded and the fibrin indicator gel was stained with 0.1% (w/v) amido black in 70% methanol and 10% acetic acid for 1 min, followed by de-staining for 1 h in two-changes of 70% methanol and 10% acetic acid without amido black.

### siRNA Treatment

Custom anti-urokinase and matched control-scrambled siRNA sequences having the same oligonucleotide composition, respectively corresponding to mRNA sequences 5-AAGAGTCTGGTGAATCGAACT-3 (Accession # NM_174147) and 5-GCGTAAGATAGCTTATCGGAA-3, were obtained from Qiagen. In separate studies, the above urokinase-specific siRNA sequence was chemically modified using Silencer Select siRNA technology to reduce off-targeting effects [Life Technologies; [45]]. A previously published bovine-specific urokinase antisense siRNA [46] and a custom luciferase-specific siRNA corresponding to the mRNA target sequence AAGCATACTCTGCCGCAGAGT (Accession # NM_075830) were used as controls (Life Technologies). For siRNA assays, BMEC were seeded in 24-well plates for 24 h prior to transfection and treated in parallel with anti-urokinase siRNA, control-scrambled siRNA, or transfection reagent alone (mock-transfection) under the routine culture conditions described above. After 24 h, BMEC cultures were transfected with a final siRNA concentration of 40 pmol using RNAiFect Transfection Reagent according to manufacturer's instructions for a maximum of 96 h, with fresh, antibiotic-free culture medium (without phenol red) added at 0 h and 48 h post-transfection. BMEC cultures were alternatively mock-transfected by addition of transfection reagent without siRNA. Transfected and mock-transfected BMEC were cultured with *C. neoformans* strain, C23, and evaluated for cryptococcal-induced urokinase mRNA or protein expression after 0 h, 24 h, 48 h, 72 h or 96 h. RNA transfection efficiency was measured in monocultured BMEC at 7 h post-transfection as described previously [47] using Alexa Fluor 488-conjugated anti-urokinase siRNA and averaged 93.7% under the conditions described above (Fig. S1). The transfection procedure, with or without urokinase-specific or control siRNAs, minimally affected BMEC integrity, viability or confluent growth at the concentration of siRNA used in this study (Fig. S1). For these studies, endothelial cell viability was measured as described above, and cell integrity was measured by LDH assay (Clontech) as described previously [21].

### Quantitative PCR

BMEC were washed 3×5 min with HBSS and total RNA was isolated from BMEC using the Qiagen RNeasy mini kit and quantified using a NanoVue spectrophotometer (GE Healthcare). cDNA was generated from 1 µg total RNA by reverse transcription with Superscript First Strand (Invitrogen) using Superscript II reverse transcriptase (50 units/reaction) and 500 ng of random hexamers under recommended conditions. Sample cDNA together with the appropriate primer sets (below) were added to iQ SYBR Green Supermix and amplified in a Bio-Rad MyiQ Single-Color Real-Time PCR detection system. Urokinase cDNA was amplified with primer sequences 5′-TCAGCGATGCAGTTGCCCAA-3′ (forward) and 5′-AGCACCAGGGCCTTCTCTGATT-3′ (reverse). The housekeeping gene, GADPH, was used as an internal control to normalize urokinase gene expression of BMEC cultures under the treatment and control conditions used in this study. GADPH was amplified with primer sequences 5′-CAAGTTCAACGGCACAGTCAAGGC-3′ (forward) and 5′-GGTGCAGGAGGCATTGCTGACAA-3′ (reverse). The Ct of GADPH (C_tGADPH_) +/− standard deviation was 18.87+/−0.232 for all conditions of BMEC (co)culture discussed in Results. C_tUROKINASE_ was normalized by subtracting C_tGAPDH_ to obtain ΔC_t_. Urokinase and GADPH were amplified in separate but parallel reactions of individual cDNA samples. Changes in urokinase expression were evaluated according to the formula: ΔΔCt = [Control (C_tUROKINASE_ - C_tGADPH_)−Infected (C_tUROKINASE_ - C_tGADPH_)], according to a previously described protocol [48], and expressed as fold-increase (2^ΔΔCt^). ‘Infected’ indicates BMEC cultured for 12 h in the presence of *C. neoformans* or *S. cerevisiae*, as indicated in Results, under the routine culture conditions described above. ‘Control’ indicates monocultured BMEC. Melting-curve analysis verified the presence of only the desired amplicons during qPCR analysis, and the correct size of qPCR products was confirmed by agarose gel electrophoresis (data not shown).

### SDS-PAGE and Western blotting

SDS-PAGE was performed as previously described [32] using NuPAGE precast 10% Bis-Tris gels (Invitrogen). Protein from gels was transferred onto polyvinylidene difluoride (PVDF) membranes using a Novex X-Cell II blot module (Invitrogen), which were afterwards blocked with 3% BSA in PBS buffer containing 0.05% Tween 20 for 14–18 h at 4°C. The blocked membranes were incubated for 1 h at 25°C with a 1∶1000 dilution of rabbit antisera (Fitzgerald Industries) against human plasminogen, 1∶200 goat-antihuman polyclonal urokinase (Santa Cruz Biotechnologies) in PBS with 2% BSA. Blots were washed four times in antibody diluent and incubated with secondary anti-rabbit or anti-goat IgG antibody (Sigma Aldrich) conjugated to horseradish peroxidase and examined for chemiluminescence. Blots were accordingly developed in peroxide and luminal/enhancer buffers (Bio-Rad) per manufacturer's instructions and processed using an automated imager (AFP Imaging) using Kodak X-OMAT Scientific Imaging Film. Films were scanned films for signal quantification using ImageJ software (http://imagej.nih.gov/ij/). Equal loading of protein samples in polyacrylamide gels was verified by measuring the total protein content of Western blot membranes as described previously [21], which varied by less than 5% (data not shown).

### Statistical analysis

Significant differences were determined by t-test or ANOVA using GraphPad Prism version 4.0, as indicated in Results. A value of p<0.05 or less was considered statistically significant. Error bars indicate SEM using the t-test or SD using ANOVA.

## Results

### Plasminogen-to-plasmin conversion occurs on *C. neoformans* during BMEC co-culture

**Figure 1:**
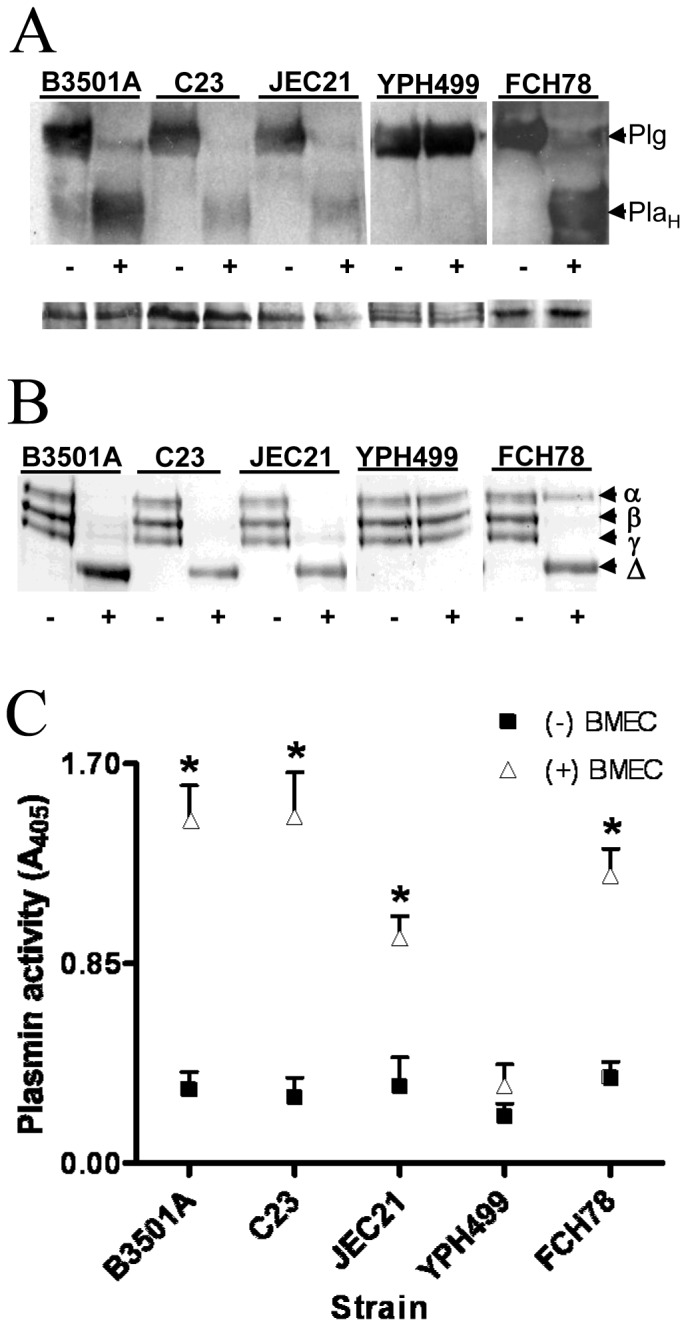
Plasminogen-to-plasmin conversion on *C. neoformans* is facilitated by exposure to BMEC. (A) Fungi were pre-coated with plasminogen, incubated in the presence (+) or absence (−) of BMEC, and analyzed by Western blot for plasminogen (Plg) or the heavy chain component (Pla_H_) of plasminogen activator-cleaved plasminogen. 50 µg protein was loaded per lane on SDS-PAGE gels, with protein loading controls shown below each blot. Representative of four experiments. (B–C) Strains were pre-coated (+) or not pre-coated (−) with plasminogen and incubated with BMEC for 12 h. Fungal cells were recovered from co-culture supernatants and analyzed by fibrin overlay zymography (B) or plasmin activity assay (C) against plasmin-specific substrates, fibrinogen (B) and Chromogenix (S-2251) (C). In (B), the α, β and γ bands of fibrinogen are indicated, with the principle degradation product labeled as Δ. Results from three experiments are shown. The Western blots shown in (A) are from separate gels processed in parallel, while the data shown in (B) are from a single gel. *p<0.05 by t-test for same-strain comparisons under the indicated conditions.

Plasminogen pre-coated strains of *C. neoformans* exhibit plasmin-dependent invasive activity in the absence of exogenous PA when cultured with BMEC [21], thus implying that the plasminogen becomes activated under these conditions. Previous studies have shown that *C. neoformans* lacks an endogenous PA activity [32]. Since BMEC secrete proteins that normally regulate the plasminogen-to-plasmin activation process, we tested whether this regulation also occurs when plasminogen is on fungal surfaces. *C. neoformans* serotype A (C23) and serotype D (B3501A and JEC21) strains and the *S. cerevisiae* control strain, YPH499, were pre-coated with plasminogen and cultured with or without BMEC in plasminogen-free assay medium. *C. neoformans* strains cultured in the presence, but not absence, of BMEC showed cleavage of surface-bound plasminogen into the heavy (63 kD) and light (26 kD, data not shown) fragments consistent with plasminogen-to-plasmin activation, as well as cleavage of the plasmin-specific synthetic substrate, Chromogenix, and the physiological plasmin substrate, fibrinogen (Figs. 1A–C). The control strain, *S. cerevisiae* YPH499, bound plasminogen but did not exhibit cleavage of surface-bound plasminogen or proteolytic activity against plasmin substrates after culture with BMEC (Figs. 1A–C), consistent with previous findings [21]. The absence of plasminogen-to-plasmin conversion on yeast surfaces in these latter experiments thus indicated the absence of YPH499-induced BMEC-secreted PA activity in culture supernatants.

Capsule expression by *C. neoformans* can promote cryptococcal-BMEC invasion by a transcellular mechanism [15], so we additionally examined whether a plasminogen pre-coated acapsular mutant elicited BMEC-regulated plasminogen-to-plasmin proteolysis. The acapsular mutant strain, FCH78, generated by insertional mutagenesis of the *CAP59* gene in strain JEC21, exhibited a BMEC-dependent cleavage of fungal-bound plasminogen and the ability to degrade plasmin-specific substrates (Figs. 1A–C). These results suggest that capsule antigens are dispensable for BMEC-mediated activation of plasminogen on *C. neoformans*.

### Conditioned medium contains PA activity

**Figure 2:**
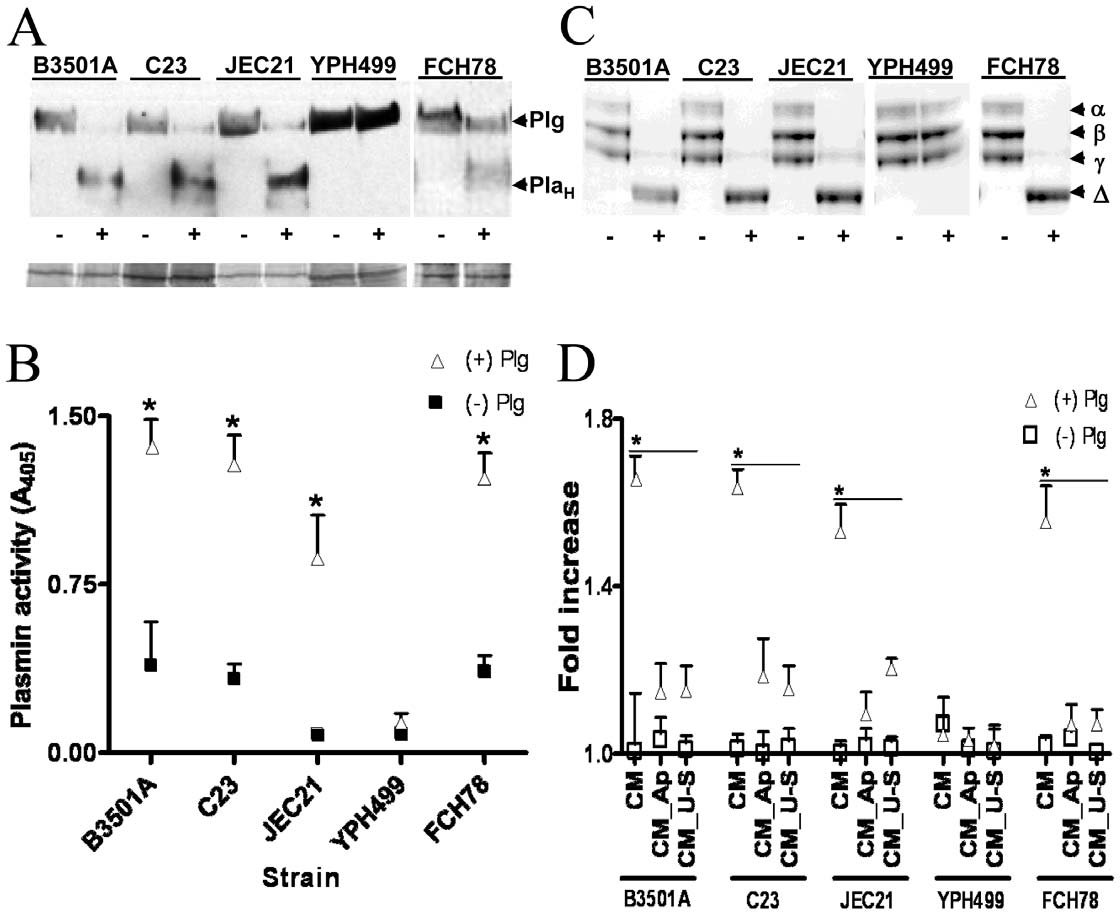
Conditioned medium from BMEC-*C. neoformans* co-cultures mediate cleavage of surface-bound plasminogen and promotes plasmin-dependent invasion of Matrigel. (A) Western blot showing the conversion of surface-bound plasminogen to its cleavage product (Pla_H_) after exposure of plasminogen pre-coated strains to conditioned medium (CM), prepared from a previous incubation with the same strain. The lower blot shows the protein load per lane of the upper blot. Representative of 3 experiments. (B–C) Surface plasmin activity against plasmin substrates, Chromogenix (B) and fibrinogen (C), of strains after CM exposure in (A). In (C), the α, β and γ bands of fibrinogen are indicated, with the principle degradation product labeled as Δ. The Western blots shown in (A) are from separate gels processed in parallel, while the data shown in (C) are from a single gel. *p<0.05 by t-test for same-strain comparisons under the indicated conditions. ‘Same-strain comparison’ refers to the comparison of activity detected when individual strains are assayed under the indicated conditions. Results from 3 experiments are shown. (D) The effect of CM on Matrigel invasion by plasminogen pre-coated (+) or non-pre-coated (−) strains. CM was prepared from 12 h cultures of each strain with BMEC or from uninfected control BMEC [CM (control)]. Invasion assays were performed with urokinase (US) or aprotinin (Ap), as indicated. The y-axis shows the number of invading fungal cells. *p<0.05 by ANOVA for the comparisons under each bar. Results from 4 experiments are shown.

To determine if plasminogen regulation on *C. neoformans* during BMEC co-culture is associated with a BMEC-secreted PA activity, we co-incubated plasminogen pre-coated strains with conditioned medium (CM) from strain-matched co-cultures. CM exposure resulted in plasminogen-to-plasmin cleavage on both hypocapsular and acapsular strains of *C. neoformans* by Western blot analysis and in fungal acquisition of plasmin protease activity against plasmin-specific substrates, Chromogenix and fibrinogen (Figs. 2A–C). Conversely, CM from BMEC-*S. cerevisiae* co-cultures lacked evidence of a soluble PA activity (Figs. 2A–C).

Plasmin-coated strains of *C. neoformans* exhibit enhanced invasion of Matrigel relative to non-plasmin coated strains [21], [32], so we additionally determined if CM exposure promoted plasmin-dependent cryptococcal invasion activity and whether PA-specific inhibitors affected this activity. Plasminogen pre-coated *C. neoformans* strains treated with CM from strain-matched fungal-BMEC co-cultures prior to invasion assays showed a nearly 2-fold increase in invasive ability over strains not pre-exposed to CM, while the control strain, *S. cerevisiae* YPH499, showed an equivalent invasive ability regardless of CM pretreatment. Plasmin-dependent Matrigel invasion activity was equivalent among acapsular strain FCH78 and hypocapsular strains of *C. neoformans*, including the JEC21 parental strain, indicating that capsule expression is not essential for this process, consistent with previous findings [21], [32]. The serine protease inhibitor, aprotinin, a potent inhibitor of plasmin protease activity, abrogated the invasive ability of CM pre-treated *C. neoformans* (Fig. 2D). Because urokinase is an established mediator of plasmin-facilitated tissue invasion, we further examined whether the urokinase inhibitors, UPA-STOP and amiloride, affected the plasmin-dependent cryptococcal invasion of Matrigel (Fig. 2D, data not shown). Both urokinase inhibitors negated plasmin-enhanced cryptococcal invasion, thus implicating urokinase in BMEC-dependent regulation of plasminogen activation on *C. neoformans* (Figs. 2A–C).

### In situ zymography and immuno-isolation of urokinase from CM

**Figure 3:**
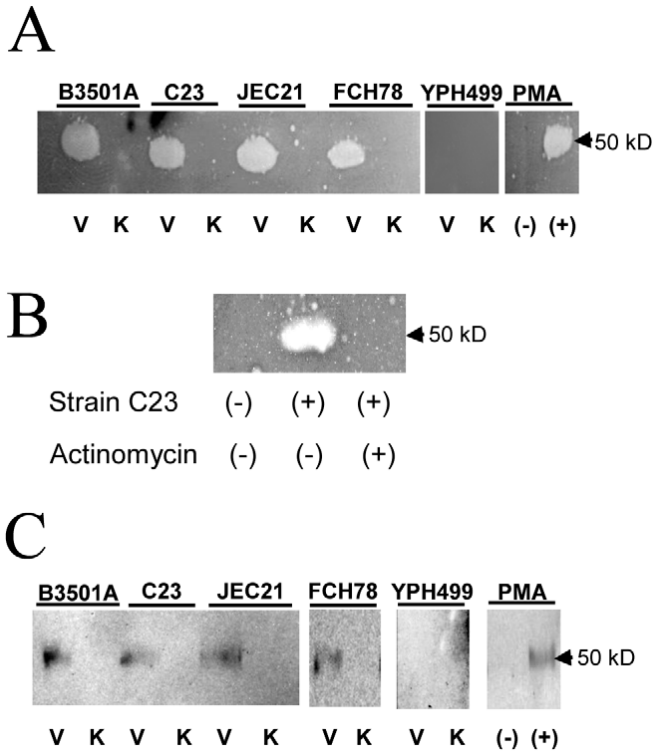
Characterization of plasminogen activator activity in CM. (A) CM was isolated from BMEC cultured with viable (V) or chemically-killed (K) strains of *C. neoformans* or *S. cerevisiae* (YPH499) and examined for PA activity by fibrin overlay zymography. Monocultured BMEC incubated in the absence (−) or presence (+) of PMA were used to control for the presence of urokinase in CM, as indicated. The clear zones result from PA-mediated plasminogen-to-plasmin conversion, which is followed by plasmin fibrinolysis. (B) BMEC were treated with the transcriptional inhibitor, actinomycin D, or mock-treated prior to culture with *C. neoformans* strain C23 and analyzed as in (A) for PA activity. (C) CM from the indicated BMEC-fungal co-cultures was immunoprecipitated with urokinase-specific polyclonal antibody. The Western blot shows the urokinase-specific band migrating at 50 kD. CM from PMA stimulated monocultured BMEC was used as positive control. The data shown in (A) and (C) are from separate gels processed in parallel. Representative of three experiments.

Plasmin(ogen)-specific fibrin overlay zymography was used to determine the molecular weight of the soluble PA activity present in CM from cryptococcal-BMEC co-cultures. In this assay, the presence of PA in samples results in the proteolytic activation of plasminogen within gels and subsequent plasmin fibrinolysis, which is detectable as clear, fibrin-free zones on Coomassie-stained gels. Overlay of plasminogen/fibrin-agarose gels with SDS-PAGE-fractionated CM from BMEC cultured with viable, but not formaldehyde- or sodium azide-killed, *C. neoformans* resulted in clear zones of plasmin-fibrinolytic activity migrating at 50 kD, the expected size of urokinase (Fig. 3A). A comparable plasminogen proteolytic activity was present in CM from monocultured BMEC treated with PMA, a known inducer of PA expression in endothelial cells [43]. CM from monocultured BMEC not treated with PMA or YPH499-BMEC co-cultures lacked detectable PA activity (Fig. 3A). BMEC pretreatment with the transcription inhibitor, actinomycin D, prior to culture with viable *C. neoformans* resulted in the loss of PA-mediated plasmin fibrinolysis in CM from BMEC-*C. neoformans* co-cultures, suggesting that induction of gene transcription is essential for PA secretion in response to viable *C. neoformans* (Fig. 3B).

This soluble BMEC-expressed PA activity was confirmed as urokinase in immunoprecipitation experiments. Urokinase was immuno-isolated from the CM of BMEC cultured with viable, but not chemically-killed, *C. neoformans* as a single band migrating at 50 kD (Fig. 3C), the same molecular weight of the PA species detected by fibrin overlay zymography (Figs. 3A–B). Urokinase was similarly purified from the CM of PMA-stimulated, monocultured BMEC, consistent with the PA activity detected on fibrin zymograms (Fig. 3A). Urokinase could not be immunoprecipitated from the CM of BMEC cultured with chemically-killed *C. neoformans* or *S. cerevisiae* strain, YPH499, or CM from monocultured BMEC without PMA (Fig. 3C). However, it is important to note that while chemical (formaldehyde or sodium azide) killing of *C. neoformans* efficiently abrogated urokinase induction in our studies, the efficacies of other methods of killing (heat, UV, etc.) have not been examined.

### *C. neoformans* induces urokinase transcription in BMEC

**Figure 4:**
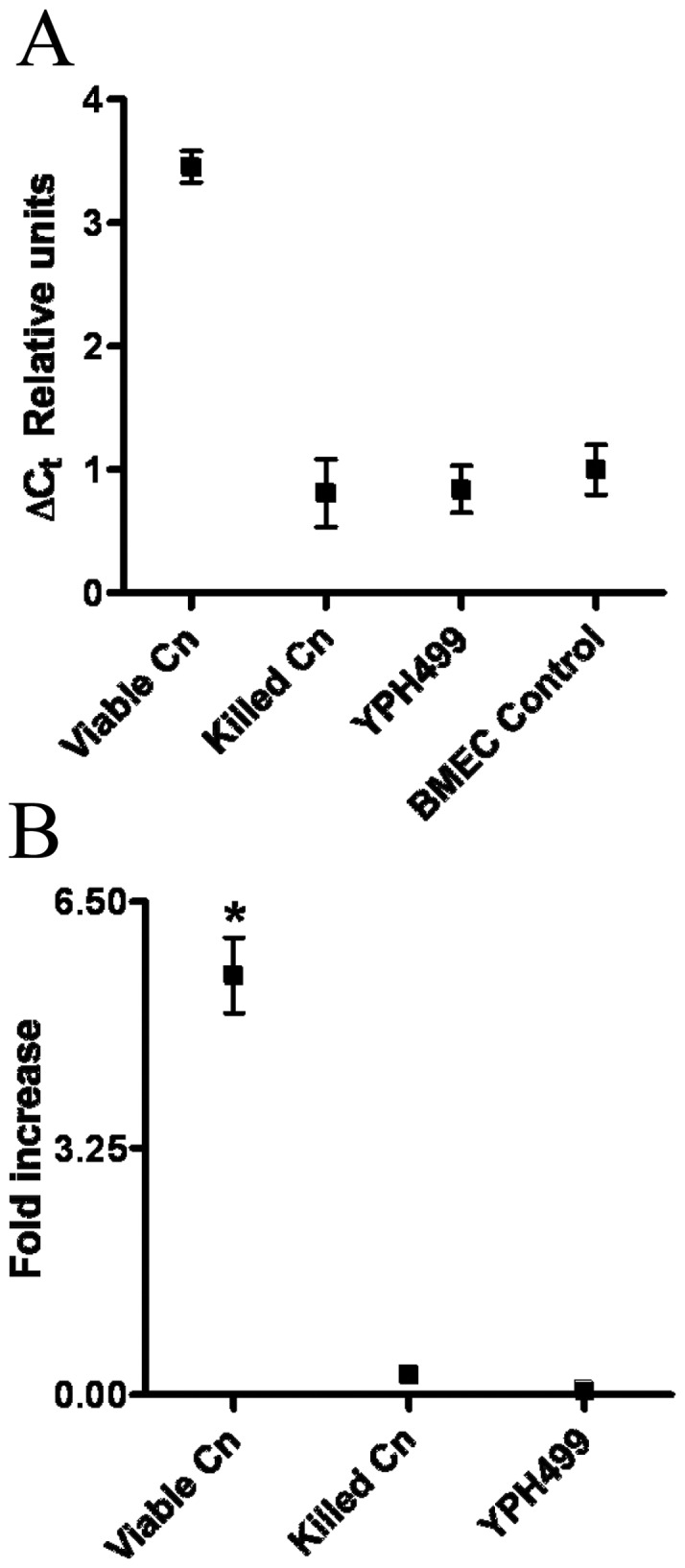
Viable *C. neoformans* induces BMEC urokinase transcription. BMEC were cultured 12 h with viable (v) or chemically-killed (k) yeast forms of *C. neoformans* (Cn) strain C23 or viable *S. cerevisiae* control strain, YPH499, followed by quantification of urokinase gene expression by qPCR. (A) The ΔCt for BMEC urokinase expression after co-culture with the indicated organisms (x-axis). The threshold of urokinase expression after GAPDH normalization (ΔC_t_) is indicated in relative units on the y-axis, which denotes increases in gene expression from low (1) to high (4) expression. (B) The fold-increase in BMEC urokinase gene expression under the indicated culture conditions relative to monocultured BMEC used as a reference. Results from 4 experiments are shown. Cn, *C. neoformans* strain C23. *p<0.05 by ANOVA.

Because urokinase is present in CM of BMEC cultured with *C. neoformans*, but not *S. cerevisiae*, and its expression is sensitive to actinomycin D, we sought to determine if *C. neoformans* modulates BMEC urokinase gene expression. Urokinase gene transcription was examined after BMEC culture in the absence or presence of *C. neoformans* strain, C23, or the *S. cerevisiae* control strain, YPH499. Results from qPCR performed on control monocultured BMEC not exposed to *C. neoformans* or *S. cerevisiae* showed that urokinase was constitutively transcribed in BMEC. Figure 4A indicates the constitutive level of BMEC urokinase transcription in relative units determined from the GADPH-normalized thresholds of gene expression activity. BMEC cultured with chemically-killed *C. neoformans* strain C23, or viable *S. cerevisiae* strain, YPH499, had no effect on the constitutive levels of urokinase transcription observed in the BMEC control. However, BMEC cultured with viable *C. neoformans* strain C23, showed an increase in C_t_ of 2.3 relative units, corresponding to an approximately 5.5-fold increase in urokinase expression over monocultured BMEC (Figs. 4A–B).

### *C. neoformans* enhances cell-associated urokinase expression by BMEC

**Figure 5:**
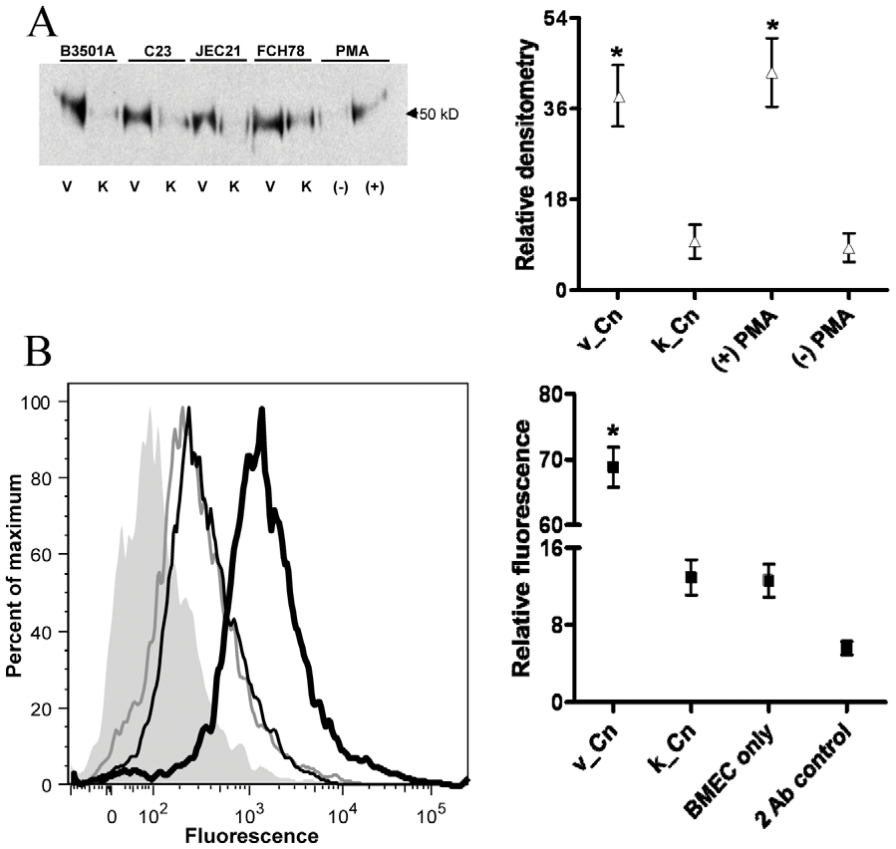
Urokinase expression on BMEC is increased after co-culture with *C. neoformans*. (A) Left panel: Representative Western blot showing cellular urokinase expression after immunoprecipitation from BMEC cultured with viable (V) or chemically-killed (K) strains of *C. neoformans* (Cn), PMA (+) or buffer only [(−); mock PMA treated]. Right panel: Relative densitometry was determined for three Western blots (left) as the percent signal distribution per blot (right). (B) Left panel: histogram showing cell surface-associated urokinase expression on BMEC after culture with killed (thin black line) or viable (thick black line) *C. neoformans* strain, C23, stained with urokinase-specific antibody. Control populations include monocultured BMEC stained with urokinase-specific antibody (thin gray line) or BMEC cultured with viable strain C23 but stained only with secondary antibody (shaded). Right panel: the relative fluorescence is shown for the indicated sample groups. Relative fluorescence is defined as the percent total distribution of mean fluorescence intensity among the indicated sample groups. *p<0.05 by t-test for BMEC urokinase expression after BMEC culture with viable versus killed *C. neoformans* (A and B) or with/without PMA (A).

Because urokinase associates with BMEC and other mammalian cells through specific receptors [23], we examined whether urokinase was present on the cell surface of monocultured BMEC and whether urokinase expression was increased on BMECs during culture with *C. neoformans*. We first used immunoprecipitation and Western blot analysis to examine the effect of *C. neoformans* on BMEC urokinase expression in whole cell lysates (Fig. 5A). Urokinase was detectable in monocultured BMEC as a faint band on Western blots, indicating minimal expression. However, a significant increase in cellular urokinase occurred following BMEC co-culture with viable, but not chemically-killed, *C. neoformans*, and a comparable increase in urokinase expression was observed in monocultured BMEC in response to PMA (Fig. 5A). The mean density of BMEC urokinase expression elicited by viable *C. neoformans* or PMA comprised 38.6% and 43.3% of the total urokinase signal density of each experimental group included in Western blots (Fig. 5A). The cryptococcal-induced increase represented a 4-fold induction over that measured in response to chemically-killed *C. neoformans*, which exhibited no increase in cellular urokinase expression relative to monocultured BMEC controls (Fig. 5A).

Single cell measurements potentially provide a more precise measure of protein quantification relative to the population-based averages obtained by SDS-PAGE/Western blot analysis [49]. We therefore used flow cytometry to quantify urokinase expression on BMEC cultured with viable or killed yeast forms from *C. neoformans* strain C23. BMEC urokinase expression after culture with chemically-killed *C. neoformans* was equivalent to that of monocultured BMEC controls and represented 12–13% of the cumulative mean fluorescence per experimental group (Fig. 5B). By contrast, the mean fluorescence of BMEC urokinase after culture with viable *C. neoformans* comprised approximately 69% of the cumulative fluorescence per experimental group and represented a 5-fold increase in urokinase expression over the constitutive levels observed in monocultured BMEC. This fold-increase in BMEC urokinase expression correlated well with the 4-fold increase detected by Western blot analysis, considering a potential loss in cell number of 5–20% on the basis of anomalous forward- and/or side-scatter profiles by flow cytometry. This 4- to 5-fold increase in urokinase protein expression is, in turn, consistent with the cryptococcal-induced 5.5-fold increase in urokinase mRNA expression by qPCR analysis (Fig. 4B).

**Figure 6:**
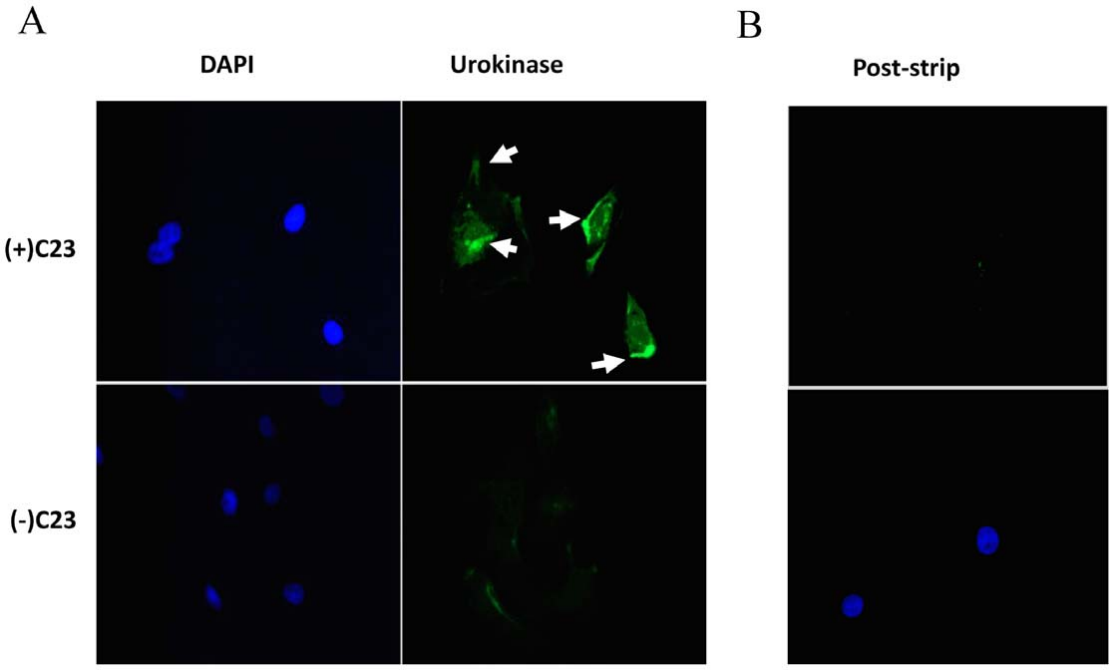
*C. neoformans* induces a heightened, polarized expression of urokinase on BMEC. (A) BMEC were cultured in the presence (+) or absence (−) of *C. neoformans* strain, C23 and afterwards washed, fixed, and analyzed for cell surface-bound urokinase (right) by indirect immunomicroscopy. Cells were co-stained with DAPI (left) to indicate the position of cell nuclei. Arrows indicate regions of urokinase accumulation along cell borders. (B) BMEC that were cultured with strain C23 and stained for cell surface urokinase expression were afterwards stripped of surface-bound urokinase and re-stained for urokinase expression (top). Cells were co-stained with DAPI (bottom). The arrowheads indicate peripheral regions of urokinase accumulation. Representative of three experiments.

The topography of urokinase expression on the surface of BMEC was next assessed by indirect immunofluorescence microscopy. Monocultured control BMEC exhibited a minimal, urokinase-specific signal that was evident as discrete clusters of fluorescence asymmetrically distributed on cell surfaces. BMEC cultured with viable *C. neoformans* strain C23, exhibited marked increases in surface fluorescence that was asymmetrically distributed, as observed with control cultures (BMEC not exposed to *C. neoformans*), but with pronounced focal accumulations of signal toward one end of the cell (Fig. 6A). A disruption of BMEC monolayer integrity was observed after BMEC incubation with *C. neoformans* and is possibly related to the ability of *C. neoformans* to damage host cells, as previously described [69]. While some of cells shown in Figure 6A are juxtaposed in the DAPI panels, they are clearly distinguishable due to the focalized, intense staining of urokinase at cellular borders. Urokinase-specific staining was confirmed by BMEC treatment with an acidic glycine buffer solution shown to effectively dissociate urokinase from cell surfaces [42]. In these control experiments, BMEC were first confirmed for cell surface urokinase expression and afterwards acid-treated and re-stained for the presence of urokinase. Acid-treatment of BMEC from *C. neoformans* co-cultures or monocultured controls exhibited loss of urokinase-specific surface fluorescence (Fig. 6B, data not shown). No fluorescence was detected in BMEC cultures stained with secondary antibody alone (data not shown).

### BMEC urokinase expression facilitates plasmin-enhanced cryptococcal-BMEC invasion

**Figure 7:**
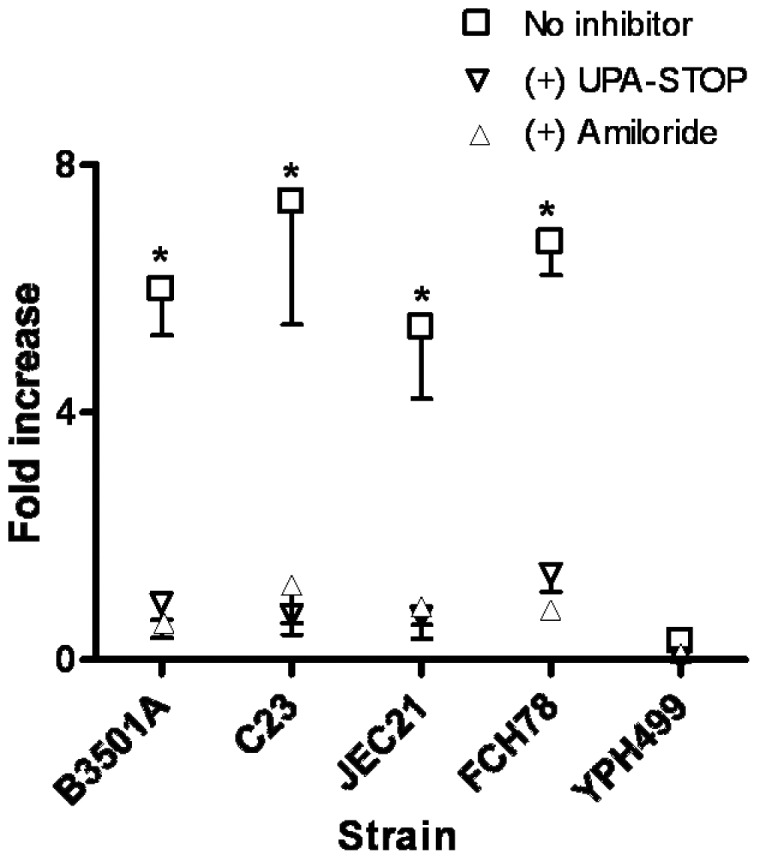
BMEC urokinase activity facilitates invasion by plasminogen pre-coated *C. neoformans*. *C. neoformans* or *S. cerevisiae* strain, YPH499, were pre-coated with plasminogen and assayed for invasive activity in a transwell BBB model system. BMEC invasion of the indicated fungal strains is calculated as the fold-increase (ratio) in invasion ability in the absence versus presence of urokinase-specific inhibitors UPA-STOP or amiloride. Results from four experiments are shown, with *p<0.05 by ANOVA for same-strain comparisons under the indicated culture conditions.

We examined whether BMEC urokinase expression induced by *C. neoformans* was sufficient to facilitate the plasmin-dependent invasive ability of plasminogen pre-coated *C. neoformans* in a transwell BBB model system. Confluent BMEC monolayers were developed in transwell inserts prior to invasion assays and expressed high barrier resistance by TEER analysis and dextran exclusion in paracellular permeability assays (Fig. S2, data not shown). The *S. cerevisiae* control strain, YPH499, showed minimal invasion activity and no relative increase in invasion when coated with plasminogen prior to invasion assays. By contrast, cryptococcal-BMEC invasion was significantly greater for plasminogen pre-coated strains than strains not pre-coated with plasminogen (Fig. 7), consistent with the augmentation of cryptococcal invasion by BMEC-expressed urokinase. The addition of the urokinase-specific inhibitors, UPA-STOP or amiloride, negated the plasmin-enhanced invasive ability of *C. neoformans*, suggesting that BMEC urokinase expression is essential for invasion (Fig. 7, data not shown). The general serine protease inhibitor, aprotinin, which is capable of targeting both urokinase and plasmin, yielded a similar inhibitory effect (Fig. 7).

### siRNA anti-sense silencing of BMEC urokinase gene expression inhibits *C. neoformans* invasion activity

**Figure 8:**
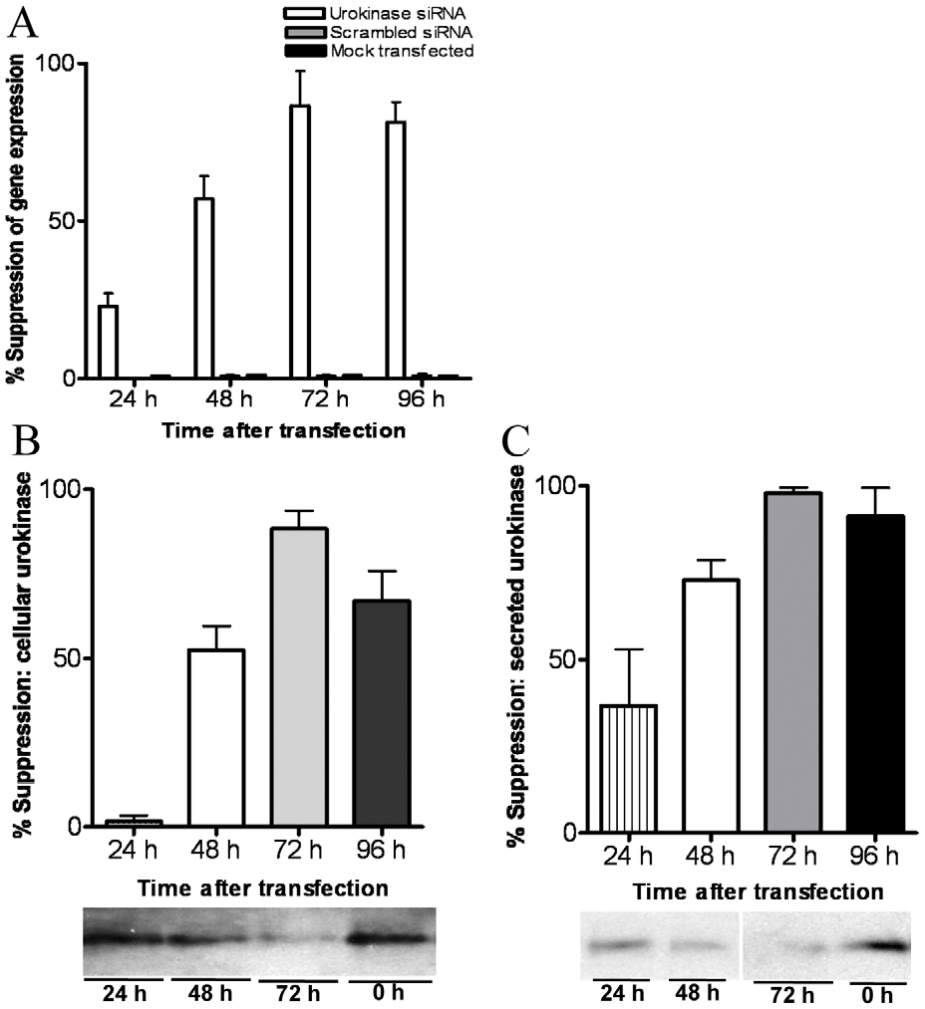
siRNA-mediated silencing of cryptococcal-induced urokinase gene expression in BMEC. (A) BMEC were treated with siRNA for the indicated times with *C. neoformans* strain C23 added to BMEC cultures 12 h prior to RNA isolation and qPCR analysis. (B–C) BMEC were analyzed by immunoprecipitation to determine cell-associated urokinase expression (B) and secreted urokinase activity present in CM (C), with representative Western blots located below each graph. Results from three experiments are shown.

We used siRNA technology to examine whether selective inhibition of *C. neoformans*-induced BMEC urokinase expression impeded plasmin-dependent cryptococcal-BBB invasion. The efficacy of siRNA-mediated silencing of *C. neoformans*-induced urokinase gene expression was first confirmed at both the mRNA and protein levels. BMEC were treated up to 96 h with urokinase-specific or scrambled siRNA prior to culture with *C. neoformans* to examine the specificity of gene silencing in BMEC-cryptococcal co-cultures and its effect on cryptococcal-BMEC invasion activity. We noted that the effect of urokinase-specific siRNA treatment was time dependent, with an average of 23% and 57% inhibition at 24 h and 48 h, respectively, and an average maximum inhibition of 87% at 72 h post-treatment (Fig. 8A). Urokinase protein levels generally declined at a similar rate to mRNA levels, but noticeable differences were observed in the initial decline of soluble versus cell-associated protein fractions over the first 48 h of treatment (Figs. 8B–C). For example, the soluble fraction of urokinase recovered from CM was reduced by an average of 37% after 24 h post-treatment with urokinase-specific siRNA, while the decline of the cell-associated fraction of urokinase over the same period was negligible (<5%). By 48 h post-treatment the average expression of soluble and cell-associated fractions of urokinase was inhibited by 73% and 52%, respectively, and maximally inhibited after 72 h at 96% (soluble) and 81% (cell-associated) (Figs. 8B–C). No suppression of urokinase gene or protein expression was observed in BMEC treated with a scrambled siRNA sequence or after mock-transfection (Fig. 8A, data not shown).

Urokinase gene expression was not measurably affected by a scrambled siRNA having the same nucleotide composition as the urokinase-specific siRNA sequence, indicating that the observed knockdown of urokinase expression in these studies is due to the selective, siRNA-directed elimination of urokinase transcripts. To further examine the potential for off-target gene silencing in our studies, the urokinase-specific siRNA sequence used in Figure 8 was chemically modified to reduce off-targeting effects and examined for the ability to inhibit urokinase expression relative to that observed with a previously published bovine-specific urokinase siRNA sequence and an irrelevant, luciferase-specific siRNA. As shown in Figure S3, urokinase-specific siRNA sequences effectively silenced BMEC urokinase gene expression in response to *C. neoformans*, thus supporting the targeting specificity of siRNA-directed urokinase gene knockdown observed in our studies, consistent with findings of other investigators [71] concerning the anti-urokinase α3 sequence shown in Figure S3.

**Figure 9:**
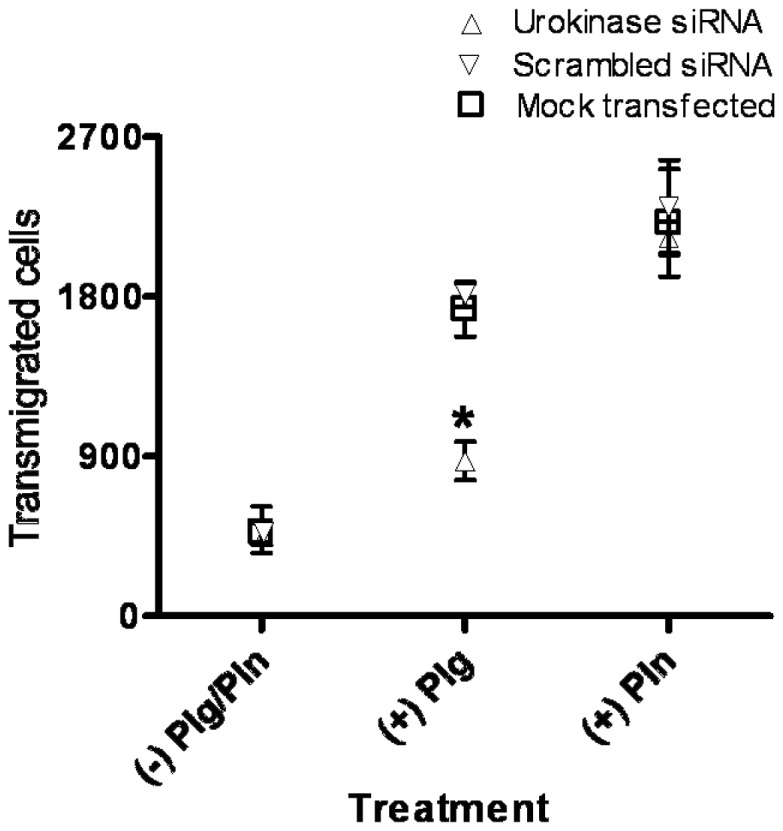
Urokinase-dependent invasion activity by *C. neoformans*. BMEC were treated for 72 h with the indicated siRNA species or mock-treated and subsequently used for transwell invasion assays conducted with plasminogen (Plg) or plasmin (Pln) pre-coated *C. neoformans* strain, C23, as indicated. Error bars depict standard deviation for 4 experiments for same-strain comparisons under the indicated conditions. *p<0.05 for (+) Plg and (+) Pln comparisons by t-test. Results from four experiments are shown.

We next examined the associated effects of siRNA-mediated urokinase gene silencing on cryptococcal-BBB invasion. Because *C. neoformans* can invade BMEC by mechanisms other than the plasmin/urokinase-dependent mechanism described here, siRNA experiments were performed with BMEC cultures maximally silenced for urokinase expression, without siRNA titration, to determine the relative importance of host urokinase in cryptococcal invasion. Strain C23 of *C. neoformans* was pre-coated with either the active serine protease, plasmin, or plasminogen and added to BMEC cultures pretreated 72 h with urokinase-specific or scrambled siRNA sequences or transfection buffer alone (mock-treated). Pretreatment of BMEC with urokinase-specific siRNA effectively abrogated the invasion activity of *C. neoformans* in the presence, but not absence, of plasminogen (Fig. 9). Conversely, siRNA-inhibited urokinase gene expression had no effect on the invasion activity of plasmin pre-coated *C. neoformans* (Fig. 9). Similarly, mock-transfection or transfection with scrambled siRNA had no inhibitory effect on the plasmin-dependent invasion activity of plasminogen pre-coated *C. neoformans* (Fig. 9). These results collectively suggest that cryptococcal-induction of urokinase gene expression is essential for plasmin-dependent fungal invasion of BMEC.

## Discussion

Microbial induction of the host PA, urokinase, is implicated in the pathogenesis of *H. pylori* and *B. burgdorferi* [26], [38]. For example, *H. pylori* infection of gastric mucosa was associated with enhanced urokinase expression [38] and disease progression toward a severe, pre-malignant gastritis that is indicative of poor patient prognosis [50], [51], while induction of urokinase expression in *B. burgdorferi*-BMEC co-cultures resulted in plasminogen-to-plasmin activation and enhanced pathogen invasion of an *in vitro* BBB model [26]. Our present study uncovers a similar link between the pathogenesis of *C. neoformans*, *in vitro*, and the induction of BMEC urokinase expression. Cryptococcal-BMEC interactions resulted in marked increases in host cell-associated urokinase expression and the appearance of soluble urokinase in CM. Plasmin-dependent invasion by *C. neoformans* was abrogated in the presence of urokinase specific inhibitors and required cryptococcal-induction of BMEC urokinase expression, as evidenced by the loss of plasmin-dependent cryptococcal-BMEC invasion after selective abrogation of host cell urokinase gene expression with anti-sense RNA.

**Figure 10:**
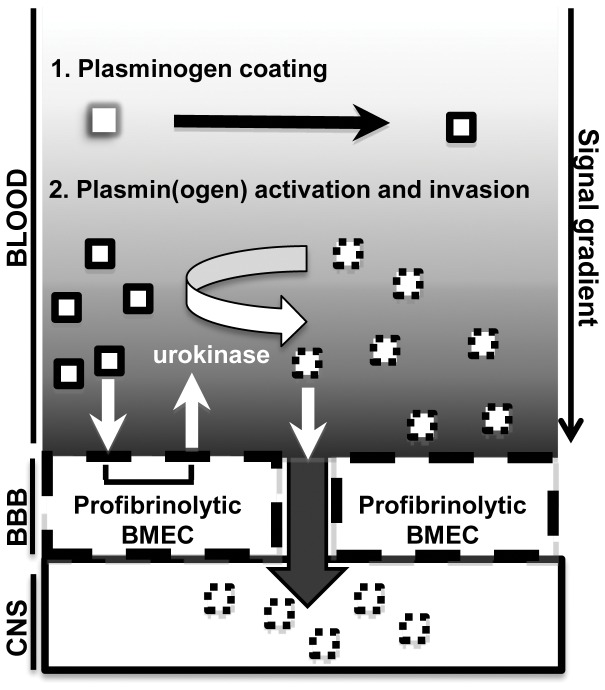
Model for urokinase-dependent, plasmin-enhanced invasion of the BBB by *C. neoformans* during the blood-borne dissemination phase of cryptococcosis. Cryptococcal yeast forms (white squares) are passively coated with plasminogen after entering the bloodstream (black border). Interactions between blood-borne *C. neoformans* and BMEC of the BBB results in the conversion of initially urokinase (−), procoagulative BMEC to a urokinase (+) profibrinolytic state. Urokinase is expressed on the BMEC surface and in the surrounding soluble milieu, which leads to plasminogen-to-plasmin activation on BMEC and fungal surfaces (dashed black border), protease degradation of endothelial junctions, and paracellular fungal-CNS invasion. The gradient-effect shown in the blood compartment reflects the relative intensity of urokinase- and plasmin-dependent signal transduction both on and in proximity to endothelial cell surfaces.

Our results show the absence of PA-facilitated plasminogen-to-plasmin activation on *S. cerevisiae* strain, YPH499, when cultured in the presence of BMEC, is due to its inability to induce host PA expression. However, when cultured with BMEC in PA-supplemented assay medium, *C. neoformans* demonstrates measurable plasmin-dependent BMEC invasion, whereas, plasminogen-to-plasmin activation is abrogated on monocultured *C. neoformans* in the absence of PA [21]. These findings suggested the BMEC-dependent regulation of plasminogen-to-plasmin activation, as observed in our experimental system, is surmounted by *C. neoformans* to promote BMEC invasion. This interpretation is supported in our current studies by the ability of *C. neoformans* to induce BMEC urokinase expression and confer a profibrinolytic phenotype on BMECs (Fig. 10).

Endothelial cells are potent regulators of plasminogen-to-plasmin activation, and this regulation can occur by multiple mechanisms including, for example, through formation of macromolecular complexes of urokinase with plasma membrane proteins and extracellular matrix components that restrict both the spatial and temporal properties urokinase activity [52]. Endothelial cells furthermore secrete PA-directed serine protease inhibitors such as plasminogen activator inhibitors 1–2 [53] and eliminate or neutralize PA through targeted cell surface receptor cleavage, and/or PA-selective membrane trafficking processes [52], [54]–[56]. Other factors that may contribute to the regulation of plasmin(ogen) in our BBB model, which consists of assay medium supplemented with 20% fetal bovine serum, include serum proteins such as α_2_ anti-plasmin that inhibit plasmin activity [57], [58]. Collectively, these processes form a regulatory barrier that prevents random activation of the plasminogen system, *in vivo*, and might curtail or prevent subversion of plasmin function, *in vivo*, by pathogen, such as *C. neoformans*, otherwise capable of advantaging the plasminogen system for host invasion, *in vitro*. However, given that our BBB model incorporates key physiological effectors and parameters of plasminogen regulation, cryptococcal ability to fundamentally alter host PA expression in this system could indicate that similar pathogen-host interactions occur during cryptococcosis. Studies examining endothelial cell regulation of fibrinolytic mediators during angiogenesis suggest that endothelial pro- and anti- fibrinolytic states are governed locally by the balance of positive and negative regulators present in the microenvironment [59]. According to this paradigm, the local induction of host urokinase expression by *C. neoformans* could drive endothelial cells toward a profibrinolytic state that actively supports plasminogen-to-plasmin activation on fungal and host cell surfaces (Fig. 10). This ‘phenotypic switch’ could explain our previous [21] and current findings regarding the potentiating effect of BMEC on plasmin-dependent cryptococcal-host cell invasion, whereas plasmin(ogen) activation and BMEC invasion are absent with *S. cerevisiae* YPH499 under similar conditions.

Based on our *in vitro* evidence, we predict that *C. neoformans* similarly modulates the fibrinolytic phenotype of BMEC during invasive cryptococcosis. Specifically, septicemia and tissue inflammation promote a procoagulative, anti-fibrinolytic vascular endothelial cell phenotype [60] that could be surmounted locally, at the interface between cryptococcal and endothelial cells (Fig. 10). For example, blood-borne *C. neoformans* can undergo prolonged interactions with the vasculature during invasive cryptococcosis [18] and, according to our above prediction, this prolonged interaction could result in the cryptococcal-induced overexpression of host urokinase, leading to the ‘phenotypic switch’ of endothelial cells from procoagulative to profibrinolytic states and a plasmin-dependent, pro-invasive microenvironment (Fig. 10).

Our findings show that *C. neoformans* induced similar fold-increases in urokinase transcription and translation, suggesting a genetic basis for the observed pathogen-driven increases in urokinase expression. An alternative, major form of urokinase regulation is mediated at the posttranscriptional level by cis-acting elements in the 3′ untranslated region (UTR) of urokinase mRNA. Adenylate/uridylate-rich instability elements (ARE's) are conserved sequences within the 3′ UTR region of urokinase mRNA targeted by trans-acting, RNA-binding proteins that govern urokinase expression by differentially regulating mRNA stability [61]–[64]. For instance, differences in urokinase expression in MDA-MB-23 cancer cells and LLC-PK_1_ porcine epithelial cells were shown to involve changes in mRNA stabilization through ARE-targeting [65]. Of particular relevance to our current studies is the apparent positive correlation between urokinase expression and mRNA stability [66], which suggests a possible basis for the low urokinase expression by monocultured BMEC controls in our study, despite constitutive urokinase transcription. Cell lines that constitutively express high levels of urokinase protein exhibit a nearly 20-fold increase in mRNA stability over cell lines with low constitutive levels urokinase expression [66]. Consequently, the *C. neoformans*-induced increases in BMEC-derived urokinase observed in our study could involve fungal modulation of both gene transcription and post-transcriptional regulatory processes.

Several surface-expressed microbial products have been implicated as inducers of urokinase gene expression in human cell lines, including outer membrane components such as bacterial lipopolysaccharides [67], [68] and proteins associated with the cytotoxin-associated antigen (cag) type IV-secretion system of *H. pylori* [25], [69]. Significantly, while hyaluronan (HA) is a ubiquitous component of host cell extracellular matrix and basement membrane protein networks, it is also synthesized by and expressed on the cell surface of *C. neoformans* [11], [15], [70] and has been shown to modulate urokinase expression in mouse macrophages [71]. In addition, interaction of the HA receptor, CD44, with low molecular weight forms of HA induces urokinase expression and facilitates urokinase-dependent tumor cell invasion, *in vitro* [72]. Cryptococcal HA is a key mediator of CD44-transduced remodeling events in plasma membrane and cytoskeletal structure that occur in connection with cryptococcal-transcytosis and fungal-CNS invasion [9]–[14], [21], [73], [74]. Other mechanisms by which pathogens alter host fibrinolytic activity have been suggested. In particular, endothelial cell damage by *Aspergillus fumigatus* is implicated in fungal induction of tissue factor [75]. Cryptococcal-dependent host damage could similarly modulate host cell fibrinolytic balance by multiple, independent signaling pathways. Damaged cells, for example, release autocrine factors such as β-fibroblast growth factor [76], [77] that can, in turn, elicit or upregulate urokinase gene transcription [78], [79]. The induction of urokinase expression in damaged cells is additionally linked to the generation of radical oxygen species [ROS; [80]–[83]], and *C. neoformans* potentially regulates ROS formation through host cell P450 enzymes [74]. In addition, cryptococcal-BMEC interactions result in the activation of the Rho GTPase-effector, P160 Rho-kinase [ROCK; [13]], and other mediators, such as protein kinase C α [PKC α; [10]], that crosstalk with urokinase induction pathways. For instance, PKC α [84] and ROCK [85] have been shown to upregulate urokinase expression by lysophosphatidic acid (LPA)-dependent or actin/microtubule-dependent pathways, respectively, and thus implicate changes in host cell lipid metabolism and/or cytoskeletal organization as potential mediators of *C. neoformans*-induced urokinase gene expression. Therefore, the ability of *C. neoformans* to modulate host cell cytoskeletal structure [10], [13], in addition to host cell plasma membrane lipid organization [9], [12], metabolism [86] and signaling [9], [12], [87], may contribute to its ability to modulate the expression and subvert the function of potent host proteases, such as urokinase.

In summary, we show that *C. neoformans* induces urokinase mRNA transcription in BMEC and that this correlates with (a) upregulation of urokinase on the BMEC surface, (b) the appearance of soluble urokinase in conditioned culture medium, and (c) marked increases in cryptococcal-BMEC invasion by a plasmin-dependent mechanism. The ability of *C. neoformans* to modulate PA expression in primary cells and use PA activity for enhanced virulence suggest that fungal subversion of the plasminogen system may contribute to invasive cryptococcosis.

## Supporting Information

Figure S1 **Transfection efficiency and associated cellular effects of siRNA transfection.** (A) Transfection efficiency was examined by quantifying cellular fluoresence after 7 h post-transfection or mock-transfection as described in Methods. Cell viability, integrity and confluent growth were examined in parallel cultures at 96 h post-transfection. Error bars depict standard error of the mean for 3 experiments per group. The vertical line indicates the siRNA concentration used in this study. (B) Alexa Fluor 488-conjugated urokinase-specific siRNA is shown in 7 h post-transfected BMEC cultures by indirect immunomicroscopy (right). Cells were co-stained with DAPI (left). Mock-transfected cells yielded no detectable fluorescent signal (not shown). Representative of four experiments.
(TIF)

Figure S2 **Comparative TEER analysis of BMEC siRNA-transfected and non-transfected BMEC.** BMEC were grown to confluence in transwell inserts and examined for their barrier activity By TEER analysis. BMEC cultures required a minimum of 4 days culture growth to reach confluence and exhibit both maximum resistance (max) indicated by the dotted line. Error bars represent mean with standard error from four experiments.
(TIF)

Figure S3 **Silencing of urokinase gene expression is unaffected after siRNA specific chemical alterations designed to minimize off-targeting effects.** BMEC were transfected with the indicated siRNA species and, 72 h post-transfection, cultured with *C. neoformans* strain, C23, for 12 h followed by immunoprecipitation of urokinase from cellular lysates. The effect of urokinase-specific siRNAs α1, α2, α3 or irrelevant luciferase-specific siRNA (control) on *C. neoformans*-induced urokinase induction was determined from parallel cultures of similarly induced, mock-transfected BMEC and quantified in relative densitometric units. α1–2 siRNAs are identical to the siRNA sequence used in Figure 8 except that the α2 sequence has been chemically modified to reduce off-targeting effects. The urokinase-specific siRNA sequence designated α3 has been shown by other investigators to effectively silence urokinase expression of bovine endothelial cells, in vitro [46]. Error bars represent mean with standard error from three experiments.
(TIF)
