# Supplementary figures and images for: Induction of Brain Microvascular Endothelial Cell Urokinase Expression by Cryptococcus neoformans Facilitates Blood-Brain Barrier Invasion
Source: PLoS One. 2012 Nov 8;7(11):e49402. doi: 10.1371/journal.pone.0049402 (PMC3493525; doi:10.1371/journal.pone.0049402)

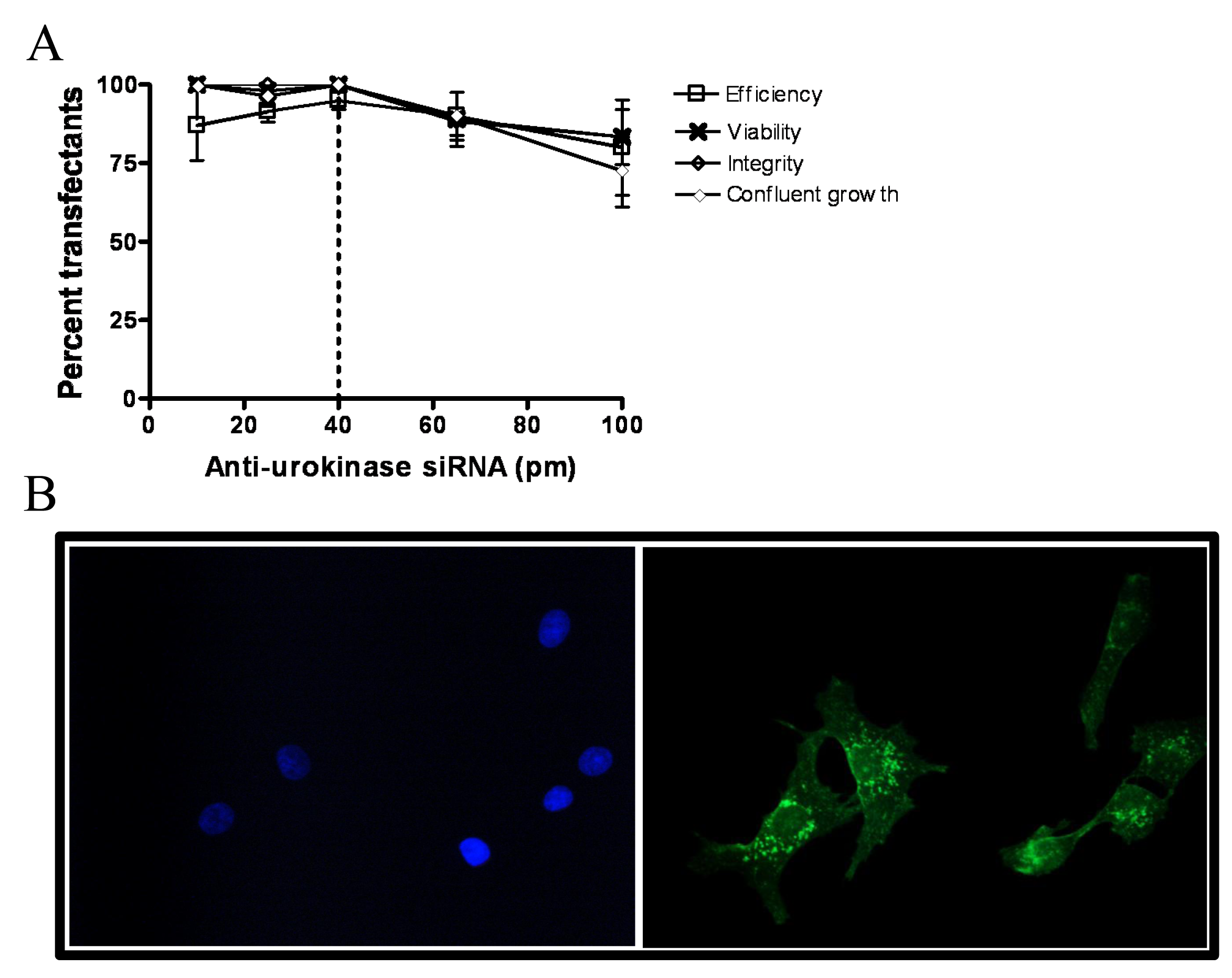

Supplement: Figure S1 — Transfection efficiency and associated cellular effects of siRNA transfection. (A) Transfection efficiency was examined by quantifying cellular fluoresence after 7 h post-transfection or mock-transfection as described in Methods. Cell viability, integrity and confluent growth were examined in parallel cultures at 96 h post-transfection. Error bars depict standard error of the mean for 3 experiments per group. The vertical line indicates the siRNA concentration used in this study. (B) Alexa Fluor 488-conjugated urokinase-specific siRNA is shown in 7 h post-transfected BMEC cultures by indirect immunomicroscopy (right). Cells were co-stained with DAPI (left). Mock-transfected cells yielded no detectable fluorescent signal (not shown). Representative of four experiments. (TIF) [file pone.0049402.s001.tif]

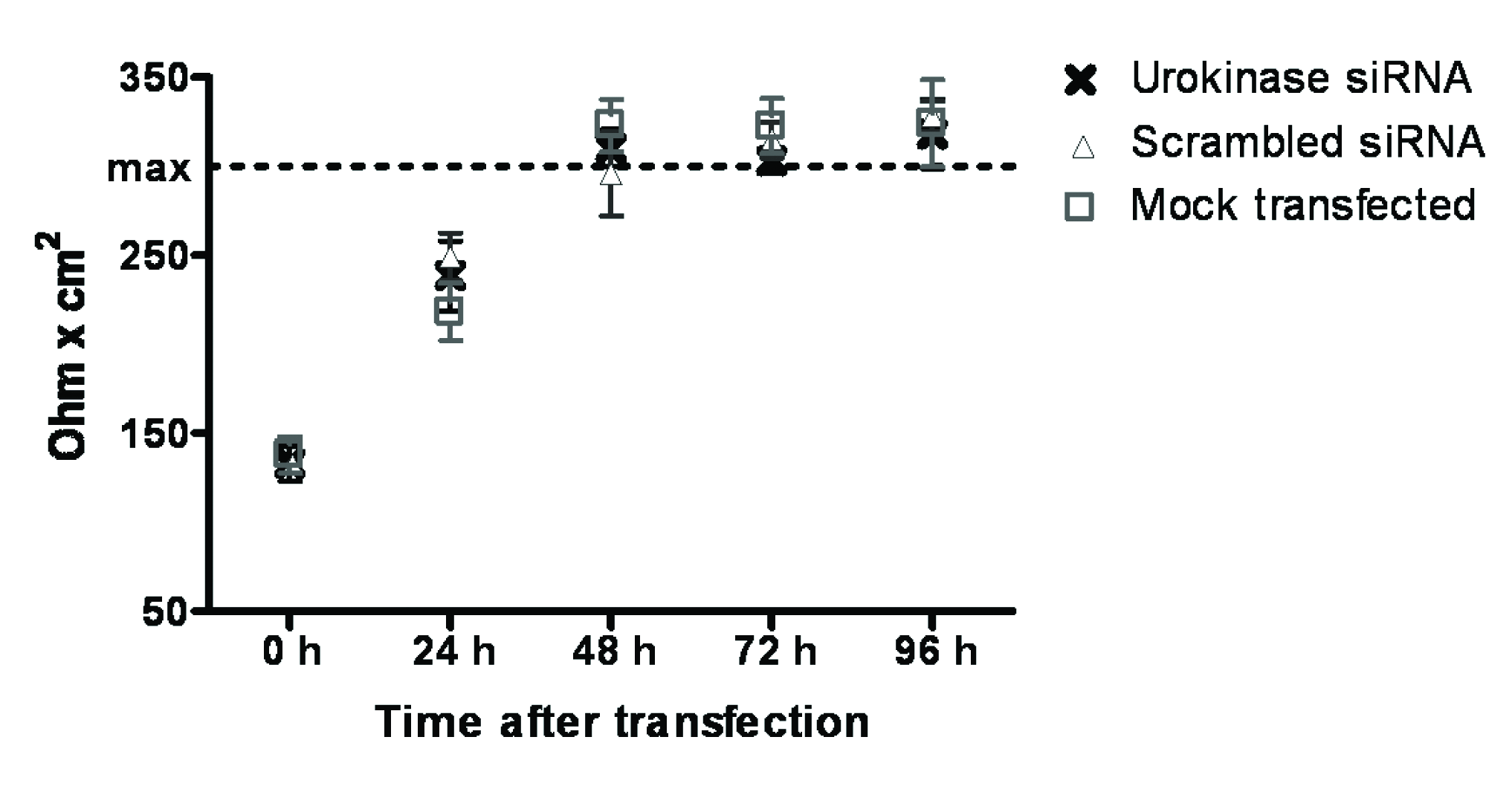

Supplement: Figure S2 — Comparative TEER analysis of BMEC siRNA-transfected and non-transfected BMEC. BMEC were grown to confluence in transwell inserts and examined for their barrier activity By TEER analysis. BMEC cultures required a minimum of 4 days culture growth to reach confluence and exhibit both maximum resistance (max) indicated by the dotted line. Error bars represent mean with standard error from four experiments. (TIF) [file pone.0049402.s002.tif]

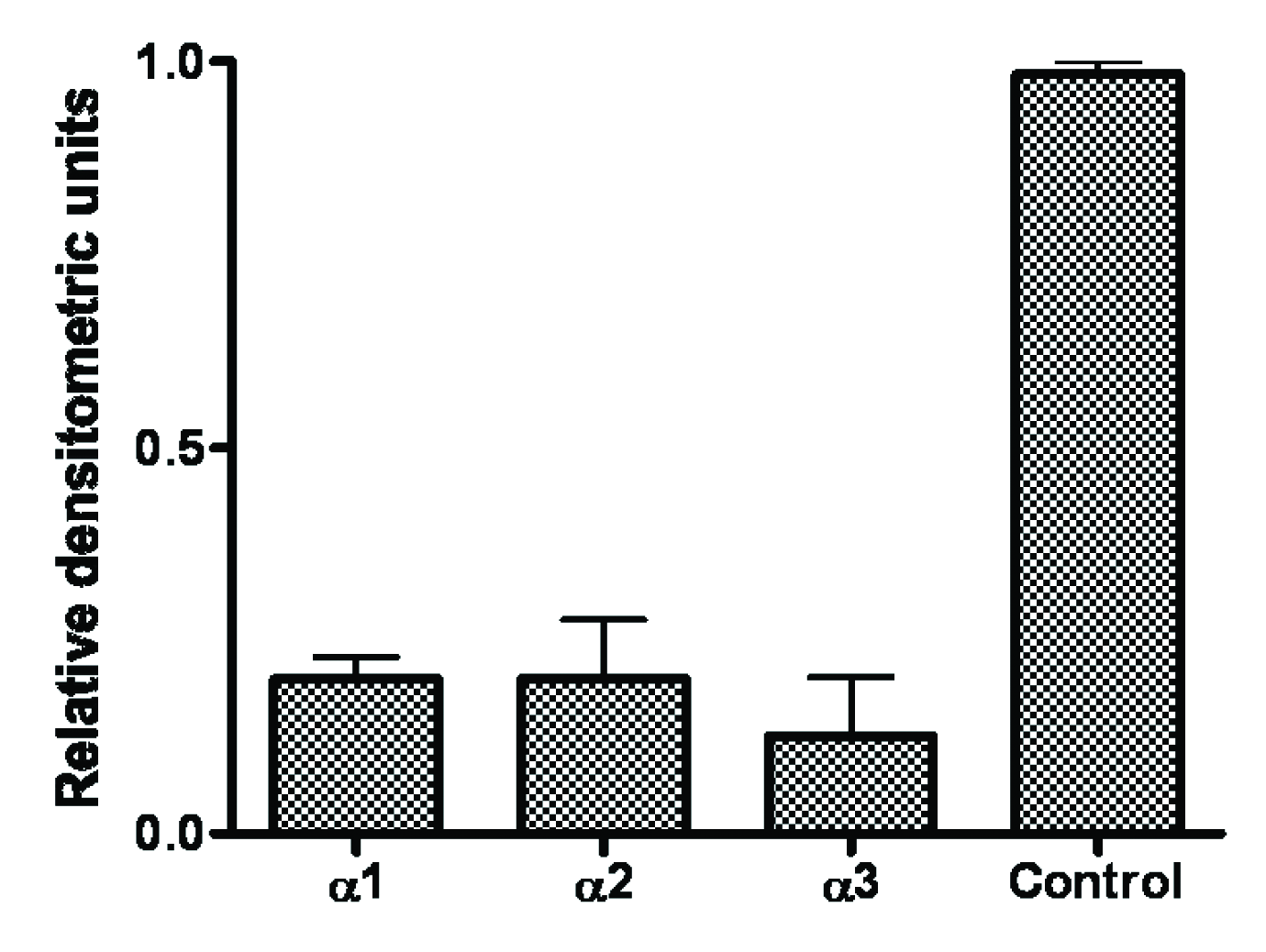

Supplement: Figure S3 — Silencing of urokinase gene expression is unaffected after siRNA specific chemical alterations designed to minimize off-targeting effects. BMEC were transfected with the indicated siRNA species and, 72 h post-transfection, cultured with C. neoformans strain, C23, for 12 h followed by immunoprecipitation of urokinase from cellular lysates. The effect of urokinase-specific siRNAs α1, α2, α3 or irrelevant luciferase-specific siRNA (control) on C. neoformans-induced urokinase induction was determined from parallel cultures of similarly induced, mock-transfected BMEC and quantified in relative densitometric units. α1–2 siRNAs are identical to the siRNA sequence used in Figure 8 except that the α2 sequence has been chemically modified to reduce off-targeting effects. The urokinase-specific siRNA sequence designated α3 has been shown by other investigators to effectively silence urokinase expression of bovine endothelial cells, in vitro [46]. Error bars represent mean with standard error from three experiments. (TIF) [file pone.0049402.s003.tif]
